# Two New Koumine-Type Indole Alkaloids from *Gelsemium elegans* Benth.

**DOI:** 10.3390/molecules18021819

**Published:** 2013-01-31

**Authors:** Mingxue Sun, Xiaoli Hou, Huanhuan Gao, Junsheng Guo, Kai Xiao

**Affiliations:** 1 Lab of Toxicology and Pharmacology, Faculty of Naval Medicine, Second Military Medical University, Shanghai 200433, China; E-Mails: sunmx@smmu.edu.cn (M.S.); houxiaoli1225@163.com (X.H.); pengyougaohuan@126.com (H.G.); 2 Department of Military Hygiene, Faculty of Naval Medicine, Second Military Medical University, Shanghai 200433, China

**Keywords:** indole alkaloids, *Gelsemium elegans* Benth., 21-oxokoumine, furanokoumine

## Abstract

Two new indole alkaloids, 21-oxokoumine (**1**) and furanokoumine (**2**), were isolated from the roots of *Gelsemium elegans* Benth together with three known compounds. The structures of the two novel compounds were elucidated by spectroscopic methods, including NMR, HR-ESI-MS, UV, IR, CD and molecular modeling. Compound **1** is the first instance of a koumine-type alkaloid with a carbonyl at the C-21 position, while compound **2** possesses a tetrahydrofuran ring located on C-20 and C-21.

## 1. Introduction

**Figure 1:**
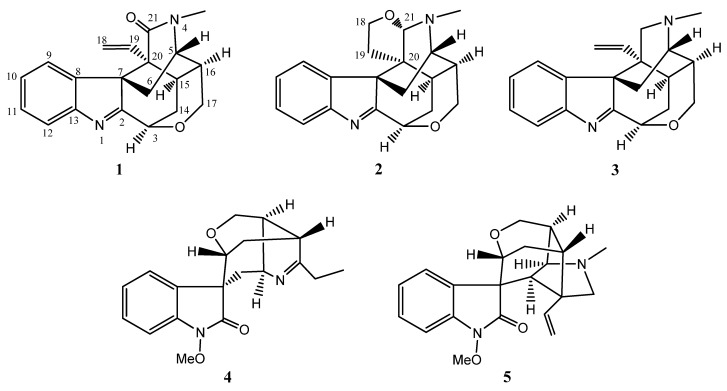
Chemical structures of compounds **1**–**5**.

*Gelsemium elegans* Benth., widely distributed in southern part of China and Southeast Asia, belongs to the family Loganiaceae and genus *Gelsemium* [1] from which more than 100 alkaloids have been reported [2,3,4,5]. Though highly toxic, *G. elegans* is still used in Chinese folk medicine for the treatment of spasticity, pain, skin ulcers, *etc.* The alkaloids from this genus are proven to possess various pharmacological effects, such as cytotoxic, analgesic, antidepressant, anti-inflammatory and antitumor activities [6]. In our studies investigating the chemical components of this plant, two new monoterpenoid indole alkaloids, 21-oxokoumine (**1**) and furanokoumine (**2**), together with three known compounds, namely koumine (**3**) [7], gelsenicine (**4**) [8] and gelsevirine (**5**) [9], were obtained (Figure 1) from the roots of *G. elegans* growing in Fujian Province in China. In this paper, the structure elucidation of the two new alkaloids is described.

## 2. Results and Discussion

**Figure 2:**
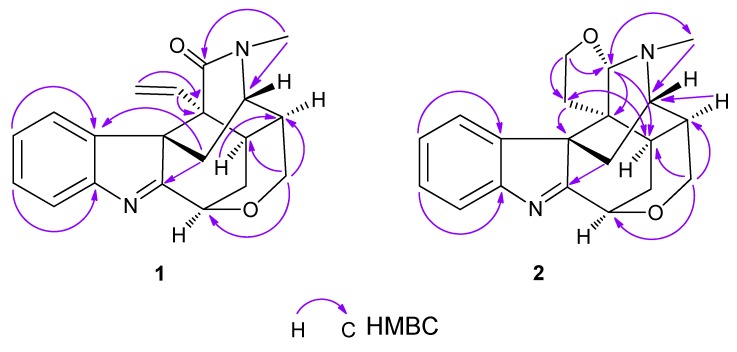
Key HMBC correlations of **1** and **2**.

Compound **1**, isolated as colorless needles, was found to possess the molecular formula C_20_H_20_N_2_O_2_ from HR-ESI-MS data [*m/z* 321.1596 ([M+H]^+^) (calcd. 321.1598)]. The UV absorptions (263 nm, 222 nm) and NMR data (Table 1) indicated that **1** was an indole alkaloid [7,10]. In the ^1^H-NMR spectrum, there existed four aromatic protons [δ_H_ 7.61 (1H, d, *J* = 7.6 Hz, H-12), 7.35 (1H, td, *J* = 7.6, 0.8 Hz, H-11), 7.25 (1H, td, *J* = 7.6, 0.8 Hz, H-10), 7.18 (1H, d, *J* = 7.6 Hz, H-9)] assignable to the indole moiety, one terminal vinyl group [δ_H_ 5.13 (1H, dd, *J* = 8.2, 4.2 Hz, H-18), 4.84 (1H, overlapped, H-19), 4.83 (1H, overlapped, H-18)], an *N*-methyl group [δ_H_ 3.2 (3H, s)], three oxygenated protons [δ_H_ 5.04 (1H, m, H-3), 4.25 (1H, dd, *J* = 12.2, 4.3 Hz, H-17), 3.69 (1H, d, *J* = 12.2 Hz, H-17)], and an aminomethine proton [δ_H_ 3.64 (1H, m, H-5)]. In the ^13^C-NMR spectrum, there were six aromatic carbon signals [δ_C_ 154.5 (C-13), 142.7 (C-8), 128.5 (C-11), 126.9 (C-10), 122.8 (C-9), 121.2 (C-12)] and an imine carbon signal [δ_C_ 183.1 (C-2)] assignable to the indole moiety, and two terminal vinyl carbon signals [δ_C_ 132.3 (C-19), 119.8 (C-18)]. The aforementioned spectral data showed a remarkable resemblance to those of koumine (**3**), which was also isolated in this study, except for the presence of a carbonyl signal (δ 171.8) in **1**, instead of a methylene signal in koumine. Based on the significant HMBC correlations between the *N*-methyl protons and the carbonyl carbon, the carbonyl oxygen was deduced to be attached to the C-21 (Figure 2). Furthermore, similar CD spectra of **1** and koumine indicated that the absolute configuration at the C-7 position was identical to that of koumine [11]. From the evidences accumulated above, the structure of **1** was established to be 21-oxokoumine (Figure 1). Compound **1** is the first instance of a koumine-type alkaloid with a carbonyl at the C-21 position.

**Figure 3:**
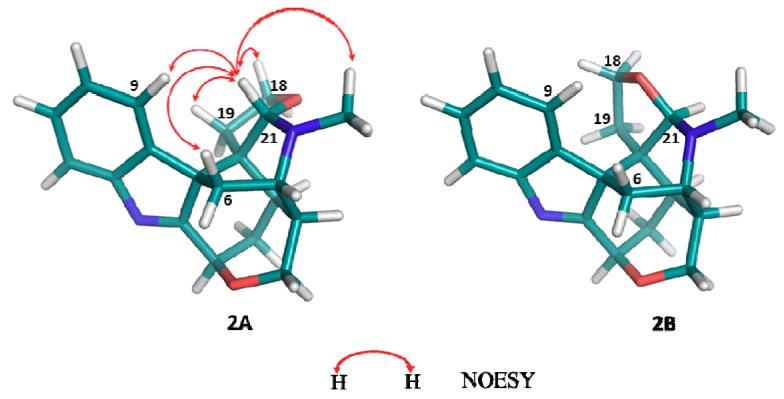
The two possible structures (**2A** and **2B**) for **2**.

Compound **2**, isolated as a white amorphous powder, was found to possess the molecular formula C_20_H_22_N_2_O_2_ from HR-ESI-MS data [*m/z* 323.1741([M+H]^+^)] (calcd. 323.1754). The UV spectrum (262 nm, 220 nm) indicated the presence of an indolenine chromophore. ^1^H- and ^13^C-NMR data (Table 1) suggested that compound **2** was a koumine-type indole alkaloid. Most of the spectral data of **2** resembles that of koumine (**3**) and 21-oxokoumine (**1**), except for the presence of two more oxygenated protons [δ_H_ 3.86 (1H, q-like, *J* = 8.0 Hz, H-18), 3.61 (1H, overlapped, H-18)] and the downfielded methine signal [δ_H_ 4.60 (1H, s, H-21), δ_C_ 98.3 (C-21)] which is connected to an oxygen atom and a nitrogen atom in contrast with the methine signal usually resonating at *ca.* 57 ppm in koumine. Furthermore, in **2**, the terminal vinyl group was substituted by two methylene groups as compared with koumine. Based on the remarkable HMBC correlations between H-18 and C-21 and C-19, between H-19 and C-7 and C-15, between H-21 and C-15, C-20 and *N*-methyl carbon, the presence of a tetrahydrofuran ring located at the C-20 and C-21, a feature that has never been found before in koumine-type alkaloids, was established (Figure 2). The significant NOESY correlations between H-21 and H-9, between H-21 and H-6*β* indicated that the three protons were on the same side of the tetrahydrofuran ring, therefore suggesting that H-21 was *β*-oriented. Considering the steric hindrance, there only exist two possible structures for **2** (**2A** and **2B**, Figure 3). Of the two optimized structures obtained by applying the molecular modeling software package SYBYL, the calculated distances between the above-mentioned proton pairs in **2A** was perfectly consistent with the NOESY data. Therefore, the orientation of H-21 was determined to be *β* and the configuration of the C-21 in **2** was determined to be *S*. From these data, the structure of **2** was elucidated to be as shown in Figure 1 and the compound was named furanokoumine.

On the basis of literature data, a plausible biogenetic pathway for the two new alkaloids was proposed. The key intermediate strictosidine, originated from tryptamine and secologanin [9], was rationalized as the precursor of monoterpenoid indole alkaloids. Strictosidine could be transformed to koumine via several steps [12], while the oxidation of C-21 of koumine could result in the formation of the new indole alkaloid **1**, and subsequent reduction of C-C bond between C-18 and C-19 would transform **1** to the new indole alkaloid **2**.

Besides these two new compounds, the structures of the three known indole alkaloids, koumine (**3**) [7], gelsenicine (**4**) [8] and gelsevirine (**5**) [9], were determined by comparing their ^1^H- and ^13^C-NMR spectroscopic data with those reported in literatures.

## 3. Experimental 

### 3.1. General

UV spectra were recorded on a CARY100 UV–Vis spectrophotometer (Varian Inc., Palo Alto, CA, USA). IR spectra were acquired using a Vector 22 Infrared spectrophotometer (Bruker Corporation, Bremen, Germany). Optical rotations were measured with a Perkin-EImer 241 polarimeter (PerkinElmer Inc., Waltham, MA, USA). CD spectrum was measured on a JASCO J-815 spectrometer (JASCO International, Tokyo, Japan). The NMR data were recorded in CDCl_3_ with TMS as internal standard on a Bruker AV-500 spectrometer instrument (Bruker Corporation) operating at 500 MHz for ^1^H and 125 MHz for ^13^C. HR-ESI-MS data were obtained by 6538 UHD Accurate-Mass Q-TOF mass spectrophotometer (Agilent Technologies, Santa Clara, CA, USA). Chromatography was performed on silica gel (200–300 mesh, Yantai Jiangyou Silica Gel Factory, Yantai, China), Sephadex LH-20 (Amersham Pharmacia Biotech AB, Uppsala, Sweden), TSK gel Toyopearl HW-40F (30–60 μm, Tosoh Co. Ltd., Tokyo, Japan) and ODS-A (50 μm, YMC, Kyoto, Japan). Analytical thin layer chromatography (TLC) was performed on HSGF 254 and the spots were detected by ultraviolet irradiation (254 and 365 nm) and by spraying with Wagner reagent. Molecular model of **2** was performed on a molecular modeling software package SYBYL (version X 1.2, Tripos Inc., St. Louis, MO, USA).

### 3.2. Plant Material

The roots of *G. elegans* Benth. (10 kg) were collected in Ningde, Fujian Province, China, in June 2009. The plant was identified by Associate Prof. Jingui Shen (Shanghai Institute of Materia Medica, Chinese Academy of Sciences) and a voucher specimen has been deposited at Lab of Toxicology & Pharmacology, Faculty of Naval Medicine, Second Military Medical University, Shanghai, China.

### 3.3. Extraction and Isolation

The air-dried roots of *G. elegans* Benth. (10 kg, dry weight) were extracted with 70% EtOH (60 L, three times under reflux). The EtOH extract (1,340 g) was suspended in H_2_O (3 L), acidified with 20% H_2_SO_4_ to pH 3, and then it was extracted with EtOAc (3 L) to remove the neutral components. The aqueous layer was extracted with CHCl_3_ (6 L) after neutralization with Na_2_CO_3_ to pH 9 to give a crude alkaloidal fraction (108 g) [10]. The fraction was separated by open-column chromatography on silica gel with a CHCl_3_/MeOH (100:0 to 50:50) gradient to give seven fractions (Fractions A–G). Fraction A (26 g) was subjected to a silica gel column chromatography eluted with cyclohexane/EtOAc/Et_2_NH (100:0:1 to 100:100:10) gradiently to give twenty subfractions (Fractions A1–A20): Subfraction A18 was further purified by ODS column chromatography eluted with MeOH/H_2_O (40:60 to 100:0). The subfraction obtained using 40% MeOH was further purified by HW-40F eluted with MeOH/H_2_O (20:80) to give **1** (3 mg). The subfraction obtained using 50% of MeOH was further purified by HW-40F eluted with MeOH/H_2_O (40:60) to afford **2** (0.9 mg). Subfraction A8 was further purified by ODS column chromatography eluted with MeOH/H_2_O (30:70 to 100:0) to furnish **3** (25 mg). Subfraction A12 crystallized to give **4** (26 mg), while subfraction A17 crystallized to afford **5** (120 mg).

### 3.4. Characterization of 21-Oxokoumine (***1***)

Obtained as colorless needles, ${\left[\alpha \right]}_{D}^{20}$: −343.2° (*c* 0.46, MeOH); UV λ_max_ (MeOH): 263 nm, 222 nm; IR (KBr) ν_max_ (cm^−1^): 3420, 2917, 1714, 1580, 1430, 1383 and 1069; HR-ESI-MS *m/z* 321.1596 (C_20_H_2__0_N_2_O_2_ [M+H]^+^, calcd. 321.1598). CD (*c* = 0.247 mmol/L, MeOH, 16 °C) Δε (λ, nm): −4.90 (268), +4.86 (228), −9.90 (202). For ^1^H-NMR and ^13^C-NMR (CDCl_3_) spectral data see Table 1.

### 3.5. Characterization of Furanokoumine (***2***)

Obtained as white amorphous powder, ${\left[\alpha \right]}_{D}^{20}$: −285.9° (*c* 0.07, MeOH); UV λ_max_ (MeOH): 262 nm, 220 nm; HR-ESI-MS *m/z* 323.1741 (C_2__0_H_2__2_N_2_O_2_ [M+H]^+^, calcd. 323.1754). CD (*c* = 0.047 mmol/L, MeOH, 16 °C) Δε (λ, nm): −24.59 (259), +29.03 (223). For ^1^H-NMR and ^13^C-NMR (CDCl_3_) spectral data see Table 1.

## 4. Conclusions

Two new indole alkaloids, 21-oxokoumine (**1**) and furanokoumine (**2**), were isolated from the roots of *G. elegans* Benth along with three known compounds. The two new alkaloids both belong to the koumine type alkaloids. A plausible biogenetic pathway for these compounds was also proposed. 

## Figures and Tables

**Table 1:** ^1^H-NMR (500 MHz) and ^13^C-NMR (125 MHz, in CDCl_3_) spectroscopic data of **1** and **2**.

	21-oxokoumine (1)		furanokoumine (2)	
Position	δ_H_	δ_C_	δ_H_	δ_C_
2		183.1		185.5
3	5.04 (1H, m)	70.7	5.02 (1H, m)	71.3
5	3.64 (1H, m)	58.3	2.88 (1H, m)	59.7
6	2.75 (1H, dd, *J* = 13.8, 3.0 Hz)	36.9	2.37 (1H, overlapped)	27.5
	2.09 (1H, *br* d, *J* = 13.8 Hz)		2.28 (1H, dd, *J* = 14.5, 3.2 Hz)	
7		57.4		57.2
8		142.7		142.1
9	7.18 (1H, d, *J* = 7.6 Hz)	122.8	7.50 (1H, d, *J* = 7.6 Hz)	123.0
10	7.25 (1H, td, *J* = 7.6, 0.8 Hz)	126.9	7.27 (1H, td, *J* = 7.6, 0.8 Hz)	126.0
11	7.35 (1H, td, *J* = 7.6, 0.8 Hz)	128.5	7.38 (1H, td, *J* = 7.6, 0.8 Hz)	128.7
12	7.61 (1H, d, *J* = 7.6 Hz)	121.2	7.62 (1H, d, *J* = 7.6 Hz)	121.5
13		154.5		155.2
14	2.61 (1H, dt, *J* = 14.7, 3.9 Hz)	25.4	2.72 (1H, dt, *J* = 14.7, 3.8 Hz)	25.8
	2.08 (1H, *br* d *J* = 14.7 Hz)		1.70 (1H, dt, *J* = 14.7, 2.2 Hz)	
15	2.71 (1H, m)	37.9	2.38 (1H, overlapped)	25.3
16	2.52 (1H, *br* d, *J* = 12.0 Hz)	31.4	2.78 (1H, m)	40.7
17	4.25 (1H, dd, *J* = 12.2, 4.3 Hz)	60.7	4.2 (1H, dd, *J* = 12.1, 4.7 Hz)	61.4
	3.69 (1H, d, *J* = 12.2 Hz)		3.62 (1H, d, *J* = 12.1 Hz)	
18	5.13 (1H, dd, *J* = 8.2, 4.2 Hz)	119.8	3.86 (1H, q-like, *J* = 8.0 Hz)	64.1
	4.83 (1H, overlapped)		3.60 (1H, dt, *J* = 4.0, 9.5 Hz)	
19	4.84 (1H, overlapped)	132.3	1.23 (1H, ddd, *J* = 12.5, 8.4 Hz)	26.6
			0.91 (1H, ddd, *J* = 12.5, 9.5, 8.0 Hz)	
20		53.9		51.2
21		171.8	4.60 (1H, s)	98.3
*N*-Me	3.20 (3H, s)	32.4	2.74 (3H, s)	41.0

## References

[1] Yamada Y., Kitajima M., Kogure N., Takayama H. (2008). Four novel gelsedine-type oxindole alkaloids from Gelsemium elegans. Tetrahedron.

[2] Dutt V., Thakur S., Dhar V.J., Sharma A. (2010). The genus Gelsemium: An update. Pharmacogn. Rev..

[3] Ouyang S., Wang L., Zhang Q.W., Wang G.C., Wang Y., Huang X.J., Zhang X.Q., Jiang R.W., Yao X.S., Che C.T. (2011). Six new monoterpenoid indole alkaloids from the aerial part of *Gelsemium elegans*. Tetrahedron.

[4] Liang S., He C.Y., Szabó L.F., Feng Y., Lin X., Wang Y. (2013). Gelsochalotine, a novel indole ring-degraded monoterpenoid indole alkaloid from *Gelsemium elegans*. Tetrahedron Lett..

[5] Zhang Z., Zhang Y., Wang Y.H., Zhang Q., Yan X.H., Di Y.T., He H.P., Hao X.J. (2012). Three novel β-carboline alkaloids from *Gelsemium elegans*. Fitoterapia.

[6] Kitajima M., Nakamura T., Kogure N., Ogawa M., Mitsuno Y., Ono K., Yano S., Aimi N., Takayama H. (2006). Isolation of gelsedine-type indole alkaloids from *Gelsemium elegans* and evaluation of the cytotoxic activity of *gelsemium* alkaloids for A431 epidermoid carcinoma cells. J. Nat. Prod..

[7] Lin L.Z., Cordell G.A., Ni C.Z., Clardy J. (1990). 19-(*R*)- and 19-(*S*)-Hydroxydihydrokoumine from *Gelsemium elegans*. Phytochemistry.

[8] Ponglux D., Wongseripipatana S., Takayama H., Ogata K., Aimi N., Sakai S.I. (1988). A new class of indole alkaloid ‘elegansamine’ constructed from a monoterpenoid indole alkaloid and an iridoid. Tetrahedron Lett..

[9] Ponglux D., Wongseripipatana S., Subhadhirasakul S., Takayama H., Yokota M., Ogata K., Phisalaphong C., Aimi N., Sakai S.I. (1988). Studies on the indole alkaloids of *gelsemium elegans* (Thailand): Structure elucidation and proposal of biogenetic route. Tetrahedron.

[10] Zhang Z., Di Y.T., Wang Y.H., Mu S.Z., Fang X., Zhang Y., Tan C.J., Zhang Q., Yan X.H., Guo J. (2009). Gelegamines A–E: Five new oxindole alkaloids from *Gelsemium elegans*. Tetrahedron.

[11] Yamada Y., Kitajima M., Kogure N., Wongseripipatana S., Takayama H. (2011). Seven new monoterpenoid indole alkaloids from *Gelsemium elegans*. Chem. Asian J..

[12] Yin S., He X.F., Wu Y., Yue J.M. (2008). Monoterpenoid indole alkaloids bearing an N4-Iridoid from *Gelsemium elegans*. Chem. Asian J..

